# Identification of DNA motif pairs on paired sequences based on composite heterogeneous graph

**DOI:** 10.3389/fgene.2024.1424085

**Published:** 2024-06-17

**Authors:** Qiuqin Wu, Yang Li, Qi Wang, Xiaoyu Zhao, Duanchen Sun, Bingqiang Liu

**Affiliations:** ^1^ School of Mathematics, Shandong University, Jinan, China; ^2^ Department of Biomedical Informatics, College of Medicine, The Ohio State University, Columbus, OH, United States

**Keywords:** DNA motifs, DNA motif pairs, chromatin interactions, TF pairs, gene transcriptional regulation

## Abstract

**Motivation:**

The interaction between DNA motifs (DNA motif pairs) influences gene expression through partnership or competition in the process of gene regulation. Potential chromatin interactions between different DNA motifs have been implicated in various diseases. However, current methods for identifying DNA motif pairs rely on the recognition of single DNA motifs or probabilities, which may result in local optimal solutions and can be sensitive to the choice of initial values. A method for precisely identifying DNA motif pairs is still lacking.

**Results:**

Here, we propose a novel computational method for predicting DNA Motif Pairs based on Composite Heterogeneous Graph (MPCHG). This approach leverages a composite heterogeneous graph model to identify DNA motif pairs on paired sequences. Compared with the existing methods, MPCHG has greatly improved the accuracy of motifs prediction. Furthermore, the predicted DNA motifs demonstrate heightened DNase accessibility than the background sequences. Notably, the two DNA motifs forming a pair exhibit functional consistency. Importantly, the interacting TF pairs obtained by predicted DNA motif pairs were significantly enriched with known interacting TF pairs, suggesting their potential contribution to chromatin interactions. Collectively, we believe that these identified DNA motif pairs held substantial implications for revealing gene transcriptional regulation under long-range chromatin interactions.

## 1 Introduction

The identification and recognition of DNA motifs binding to transcription factors (TFs) are pivotal for comprehending the regulatory mechanisms governing gene expression and cellular processes (Wong et al., 2013). A DNA motif denotes to a short, similarly repeated pattern of nucleotides that holds biological significance (Hashim et al., 2019). Deciphering these binding DNA motifs provides researchers with insights into the regulation and control of genes, fostering a deeper understanding of diverse biological phenomena (Liu et al., 2018; Yang et al., 2019; Li et al., 2024; Wang et al., 2024). With the development of high-throughput technology, several experimental techniques are available for determining TF binding DNA motifs, such as Chromatin Immunoprecipitation (ChIP) (Park, 2009), Electrophoretic Mobility Shift Assay (EMSA) (Hellman and Fried, 2007), DNA Affinity Purification Sequencing (DAP-seq) (Bartlett et al., 2017), and Systematic Evolution of Ligands by Exponential Enrichment (SELEX) (Gold, 2015). Moreover, researchers can access relevant databases to query for associated DNA motifs. For instance, JASPAR (Castro-Mondragon et al., 2022) is a widely utilized DNA motif database for storing and analyzing transcription factor binding site. TRANSFAC (Wingender et al., 2000) is a classic database containing DNA motifs of transcription factors and regulatory elements, offering a wealth of DNA motif data and associated biological information. Other databases include UniProbe, Cis-BP, motifMap, ScerTF, TFcat, and FlyTF (Fulton et al., 2009; Pfreundt et al., 2010; Daily et al., 2011; Robasky and Bulyk, 2011; Spivak and Stormo, 2012; Weirauch et al., 2014). However, the action of a single DNA motif is limited, and actual gene regulation often involves intricate interactions among multiple DNA motifs, giving rise to DNA motif pairs (Pilpel et al., 2001). These pairs of DNA motifs play a pivotal role in maintaining the accuracy and flexibility of gene expression (Clauss and Lu, 2023).

When two DNA motifs coexist and interact in a specific manner during gene regulation, they can either cooperate or compete to influence gene expression. This is pivotal for unraveling the intricate mechanisms of gene regulation networks, cell signaling, and biological processes (Kim and Wysocka, 2023). Moreover, these predictions of the interaction between DNA motifs find broad applications in bioinformatics, facilitating genome annotation and the anticipation of protein-nucleic acid interactions, thereby equipping researchers with potent tools to decipher biological data (Khodabandelou et al., 2020; Wang et al., 2022). Lastly, the underlying chromatin interactions between different DNA motifs are associated with various diseases (Bhatia and Kleinjan, 2014). Consequently, predictions based on DNA motif pairs hold promise for discovering new drug targets and innovations in the field of biotechnology, deepening our understanding of gene regulation networks (Makolo and Suberu, 2016).

The essence of DNA motif pairs lies in discerning pattern pairs, specifically identifying statistically significant pattern pairs within two correlated sequences, derived from different sequences. Current methods for identifying DNA motif pairs can be broadly classified into two types. The first approach is direct, involving the independent identification of statistically significant DNA motifs from two correlated sequences. Subsequently, the threshold is calculated to combine the DNA motifs on both sides of sequences to select statistically significant DNA motif pairs. This method may result in the exclusion of DNA motifs capable of forming pairs but are underrepresented. The second approach is based on statistical significance and involves predicting DNA motif pairs through a global optimization model. This method requires constructing a well-designed model for predicting DNA motif pairs. The algorithm developed by Ka-Chun Wong’s research group in 2016, referred to as Wong’s 2016 (Wong et al., 2016), and EPmotifPair (Wang et al., 2022) both belong to the first category of methods in existing approaches on HI-C (van Berkum et al., 2010) data for predicting DNA motif pairs. Wong’s 2016 is presently the first method for identifying DNA motif pairs on HI-C data. It can more flexibly learn sequence features in different directions, such that disturbances in predictions on one side may not affect predictions on the other side. EPmotifPair (Wang et al., 2022) predicts DNA motif pairs in a set of sequences integrated from enhancer sequences and promoter sequences. By comprehensively considering multiple co-occurring sequence patterns, it reduces the error rate compared to the separate prediction of DNA motifs. MotifHyades (Wong, 2017) belongs to the second category of methods for predicting DNA motif pairs. It adopts the probability model and utilizes two derived optimization algorithms to find DNA motif pairs with linear complexities. However, Wong’s 2016 (Wong et al., 2016) not only overlooks underrepresented DNA motifs that could have formed pairs but is also time-consuming. EPmotifPair (Wang et al., 2022) not only fails to account for potential interactions between DNA motifs but also requires the specification of numerous parameters, such as the predetermined number of DNA motifs. MotifHyades (Wong, 2017) improves the computational speed and accuracy compared with Wong’s 2016 (Wong et al., 2016), but it is sensitive to the choice of the initial value. Additionally, the probability model adopted by MotifHyades (Wong, 2017) assumes conditional independence within each sequence pair, disregarding potential interactions among DNA motifs.

**FIGURE 1:**
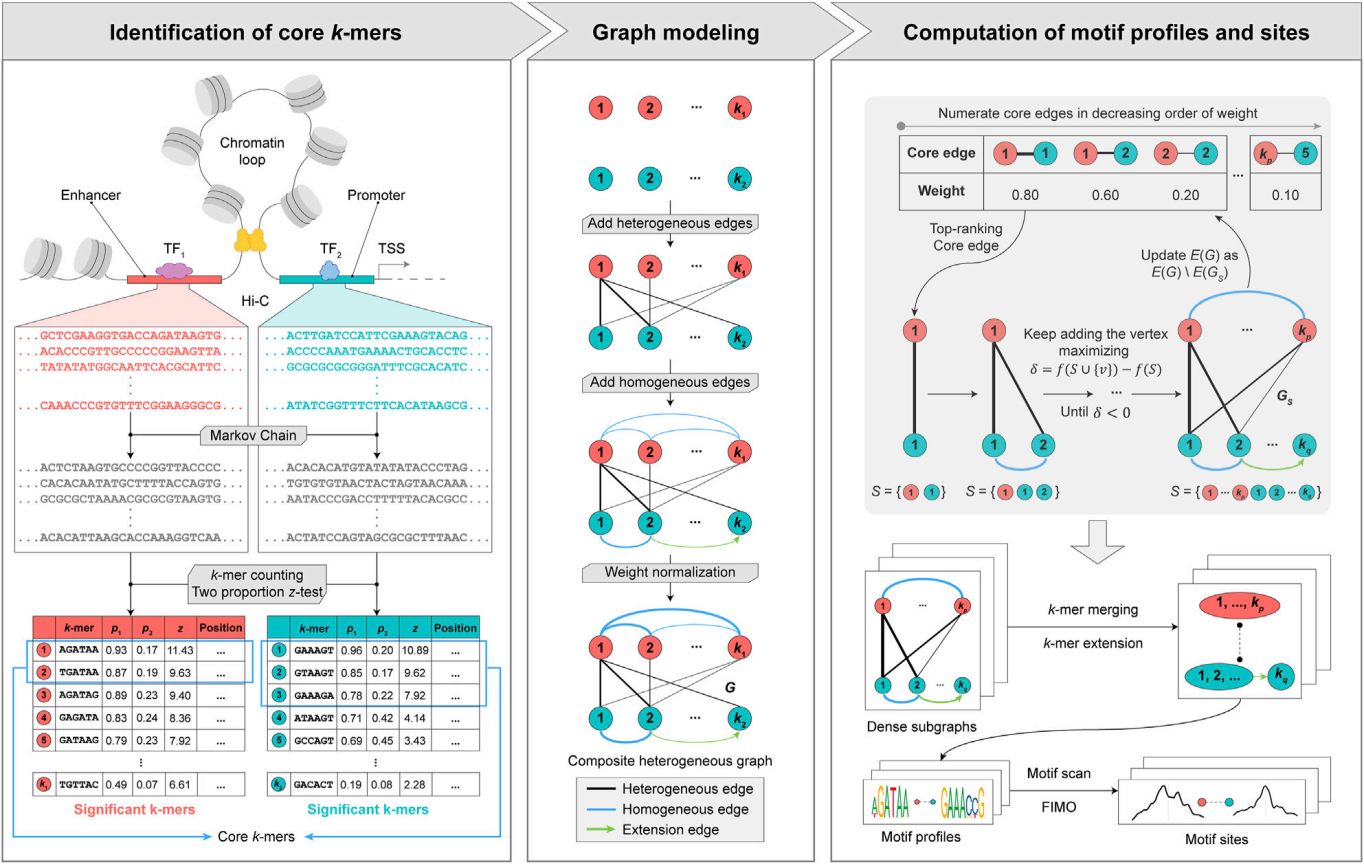
Overview of MPCHG. Enhancer (red)—Promoter (blue) interaction is used as an example for DNA motif pairs identification. p_1_ denotes the frequencies of *k*-mers in the real sequence sets and p_2_ denotes the frequencies of *k*-mers in the background sequence sets. *z* denotes *z*-score, which is used to measure the significance of *k*-mers. The *k*-mers are arranged in descending order by the size of the *z*-score. The *k*-mers framed by the blue square indicates core *k*-mers. In section Graph modeling, the black lines represent the heterogeneous edges, which connect different types of *k*-mers, and the thickness of the lines indicates the weight of the connected edges, the greater the weight, the thicker the lines. The blue line represents the homogeneous edge, and they will connect the overlapping *k*-mers, and the thickness of the lines indicates the weight of the connected edges, the thicker the line, the greater the weight of the connected edge. The green arrow is called extension edge, indicating the overlap between the two *k*-mers, which can be used to merge and extend the two *k*-mers in subsequent steps.

To address the aforementioned challenges, we propose a graph theory-based approach named MPCHG. The methodology is elucidated in Figure 1 (This paper takes Enhancer-Promoter as an example). It helps capture multiple relationships between different k -mers, including both within-sequence and between-sequence relationships. Subsequently, a community detection algorithm is employed to obtain a dense subgraph, considering not only the topology of the network but also the practical significance of node connections. Importantly, we refrain from predefining the length of DNA motifs and the number of DNA motif pairs, avoiding the loss of some important DNA motifs or the presence of high noise. We apply MPCHG to analyze seven sets of HI-C data. The results reveal a higher proportion of DNA motifs matching existing databases for predicted DNA motif pairs. The identified paired DNA motifs demonstrate higher DNase accessibility than the background sequences, and the functional consistency of DNA motifs within pairs is evident. Particularly noteworthy is the acquisition of predicted TF pairs from the predicted DNA motif pairs, and we discover that the predicted TF pairs are enriched with the interacting TFs in the STRING database. It can be seen that predicting DNA motif pairs on HI-C data can help us understand the regulatory mechanisms of genes.

## 2 Methods

### 2.1 Data collection

The input data comprises of Hi-C data from seven sets derived from six distinct cell lines, namely,: K562, GM12878, HeLa-S3, HUVEC, IMR90, and NHEK. Two sets of Hi-C data (referred to as K562_1 and K562_2, respectively) are obtained from the K562 cell line, featuring variations in data preprocessing and annotation approaches. A set of protein-protein interaction data retrieved from the STRING database (Mering et al., 2003) serves as benchmark data to assess the performance of predicted DNA motif pairs. The first set of processed Hi-C data from the K562 cell line (K562_1) is acquired from the article published by Ka-Chun Wong in 2016 (Wong et al., 2016). In this study, chromatin fragments are classified into four categories: E (Enhancer), TSS (Promoter), WE (Weak Enhancer), and PF (Promoter-Flanking Region). These categories collectively form 10 interacting pairs, resulting in a total of 74,552 long-range regulatory region pairs. The number of each interaction type is detailed in Supplementary Figure S1A. The remaining six sets of processed HI-C data are sourced from the article published by Wang in 2022 (Wang et al., 2022). Which are normalized using the Knight and Ruiz normalization vectors (Lyu et al., 2020) by Rao *et al.* (Wang et al., 2022). Notably, their chromatin interaction type is exclusively Promoter-Enhancer, in contrast to the first set of data. The long-range regulatory region pairs are summarized in Supplementary Figure S1B. In pursuit of elucidating the mechanism of DNA motif interactions, protein-protein interaction data are obtained from the STRING database, resulting in the extraction of 4,950,896 pairs of experimentally validated data. By comparing the protein names in the STRING database with transcription factors (TFs) in the JASPAR database, experimentally verified TF-TF interactions are identified, encompassing a total of 65,290 TF-TF interactions, involving 583 TFs.

### 2.2 Generation of background sequences

We utilize a third-order Markov model (Eddy, 2004) to create background sequences corresponding to each sequence (referred to as the real sequence) within the input sequence pairs. The generated background sequences are designed to align with the number and length of the given chromatin sequences, and their composition is determined by the nucleotide frequencies observed in the dataset.

### 2.3 Identification of significant k -mers

We enumerate all possible k -mers (with k=6 by default⁠) employing a sliding window approach in both the real and background sequence sets concurrently. Let $n_{F}\left(k_{i}\right)$ and $n_{B}\left(k_{i}\right)$ represent the counts of occurrences of a k -mer $k_{i}$ in the real and background sequence sets, respectively. Similarly, let $p_{F}\left(k_{i}\right)$ and $p_{B}\left(k_{i}\right)$ denote the frequency of each k -mer $k_{i}$ in the real and background sequence sets, respectively. Recognizing the reverse complementary nature of DNA, we define the frequency of a k -mer as the sum of the frequencies of the k -mer and its reverse complementary counterpart. Additionally, we exclude k -mers such as AAAAAA due to insufficient variation and discriminative power. Including them in the statistics could introduce noise and compromise the performance of the model. Assuming that the frequency distribution of k -mers follows a normal distribution, we retain k -mers with frequencies exceeding one standard deviation in the real sequences, deeming these k -mers as significant. Subsequently, we maintain the same selection k -mers in the background sequences. Following this, we use a two-proportion z-test with the null hypothesis that the frequencies $p_{F}\left(k_{i}\right)$ in the real sequence sets and $p_{B}\left(k_{i}\right)$ in the background sequence sets are the same to evaluate the significance of k -mers occurrences (Eqs 1-4):

$$H_{0}:p_{F}\left(k_{i}\right)=p_{B}\left(k_{i}\right),$$ (1)

$$H_{1}:p_{F}\left(k_{i}\right)>p_{B}\left(k_{i}\right),$$ (2)

$$z_{i}=\frac{p_{F}\left(k_{i}\right)-p_{B}\left(k_{i}\right)}{\sqrt{p_{i}\left(1-p_{i}\right)\left(\frac{1}{\sum n_{F}\left(k_{i}\right)}+\frac{1}{\sum n_{B}\left(k_{i}\right)}\right)}},$$ (3)

where,

$$p_{F}\left(k_{i}\right)=\frac{n_{F}\left(k_{i}\right)}{\sum_{j}n_{F}\left(k_{i}\right)},p_{B}\left(k_{i}\right)=\frac{n_{B}\left(k_{i}\right)}{\sum_{j}n_{B}\left(k_{i}\right)},p_{i}=\frac{n_{F}\left(k_{i}\right)+n_{B}\left(k_{i}\right)}{\sum n_{F}\left(k_{i}\right)+\sum n_{B}\left(k_{i}\right)}.$$ (4)

Where the k -mer $k_{i}$ is considered a core k -mer if it corresponds to a z -score greater than 1.96.

### 2.4 Construction of composite heterogeneous graph

We treat each k -mer as a node and construct a composite heterogeneous graph by establishing edges between them. Based on the positional information of each type of k -mer in the real sequence pairs, if two distinct types of k -mers are situated in different sequences within a sequence pair, we establish a connection between these two k -mers, referring to this connection as pair edges. The weights for pair edges are computed using Eq. 5. The first term in Eq. 5 assesses the practical significance of the edge connection between nodes $v_{i}$ and $u_{j}$ based on the number of sequence pairs they co-occur in. If they appear frequently together, the edge weight will be higher. The second and third terms in Eq. 5 consider the topological structure of the graph. They incorporate the number of neighborhoods for nodes $v_{i}$ and $u_{j}$ ​, respectively, relative to the total number of k -mers belonging to enhancers and promoters. This helps balance the importance of the nodes in the graph. Next, we introduce the concept of a neighborhood: for k -mers of the same type (promoter or enhancer), if one k -mer differs from another k -mer by only one mismatched base or has at least four consecutive identical bases, we consider the two k -mers as neighbors and establish a connection between them, denotes as neighborhood edges. The weights for neighborhood edges are determined using Eq. 6. It considers the proportion of common k -mers between nodes $v_{p}$ and $v_{q}$ ​relative to the total number of k -mers in each node. Higher weights indicate a higher similarity or overlap between the k -mers, which signifies a stronger relationship in the graph. Finally, we normalize the weights for the edges of the graph G using Eq. 7 for ensuring that the weights are scaled appropriately relative to each other. In this framework, k -mers, treated as nodes, and the interconnected edges between k -mers collectively form the weighted heterogeneous graph G .

$$\omega \left(v_{i},u_{j}\right)=\frac{N\left(v_{i},u_{j}\right)-N_{\min}\left(v_{i},u_{j}\right)}{{N_{\max}\left(v_{i},u_{j}\right)-N}_{\min}\left(v_{i},u_{j}\right)}+\frac{L\left(v_{i}\right)}{n\left(E\right)}+\frac{L\left(u_{j}\right)}{m\left(TSS\right)},$$ (5)

$$\omega \left(v_{p},v_{q}\right)=\frac{L\left(v_{p}\cap v_{q}\right)}{L\left(v_{p}\right)}+\frac{L\left(v_{p}\cap v_{q}\right)}{L\left(v_{q}\right)},$$ (6)

$$\omega^{\prime }=\frac{\omega -\omega_{\min}}{\omega_{\max}-\omega_{\min}},$$ (7)

where, $v_{i}$ is a k -mer belonging to enhancer sequences, $u_{j}$ is a k -mer belonging to promoter sequences, $N\left(v_{i},u_{j}\right)$ represents the number of sequence pairs in which $v_{i}$ and $u_{j}$ belong, $L\left(v_{i}\right)$ and $L\left(u_{j}\right)$ represent the number of neighborhoods for $v_{i}$ and $u_{j}$ separately, $n\left(E\right)$ and $m\left(TSS\right)$ represent the num of k -mers belonging to enhancers and promoters, respectively. $L\left(v_{p}\cap v_{q}\right)$ represents the num of union of $v_{p}$ and $v_{q}$ , $L\left(v_{p}\right)$ and $L\left(v_{q}\right)$ represent the num of $v_{p}$ and $v_{q}$ , separately. ω denotes the weights of edges in graph G , $\omega_{\max}$ and $\omega_{\min}$ represent the maximum and minimum weights of edges in graph G , respectively.

### 2.5 The acquisition of dense subgraphs

We apply a community discover detection algorithm to identify dense subgraphs. Firstly, we define the fitness function for evaluating the density of a subgraph. Let S be a connected subgraph of graph G , where V represents the vertex set of subgraphs S , and $E_{S}$ represents the edge set of S . Let $\left.n_{S}\left.=\right|V_{S}\right|$ and $\left.m_{S}\left.=\right|E_{S}\right|$ . By adding $\frac{n_{S}\left(n_{S}-1\right)}{2}-m_{S}$ edges to S , we form a complete graph $S^{\prime }$ , with the newly added weights are set to the average weight of graph G . The density of subgraph S is assessed by considering the difference in weights between the existing edges in S and the newly added edges. Eq. 8 outlines the community evaluation function $f\left(S\right)$ for subgraph S :

$$f\left(S\right)=\sum_{v_{i}u_{j\in E\left(S\right)}}\omega \left(v_{i}u_{j}\right)-\frac{1}{2\left|E\left(G\right)\right|}\left(n_{S}\left(n_{S}-1\right)-2m_{S}\right)∙\sum_{v_{i}u_{j}\in E\left(G\right)}\omega \left(v_{i}u_{j}\right).$$ (8)

Obviously, the larger $f\left(S\right)$ , the denser the subgraph *S* in the given sense. For a node $v\notin V_{S}$ , the fitness function $\delta_{S}\left(v\right)=f\left(S\cup \left\{v\right\}\right)-f\left(S\right)$ for v in S is defined, and the node that the maximizes fitness function, i.e., $\delta_{S}\left(v\right)>0$ , is added to the existing subgraph S .

It is worth noting that when identifying dense subgraphs, we select the point with the highest number of neighborhoods in the core pairs, possessing the highest weight, as the initial point. An iterative process ensues, continuing until no node is found that satisfies the condition, resulting in the formation of the current dense subgraph. Subsequently, we select the two nodes from the pair with the highest weight among the remaining core pairs as the initial nodes for the iterative process. Nodes that do not belong to any dense subgraph are considered isolated points and are excluded from the analysis. For the resulting dense subgraph $C=\left\{C_{1},C_{2},\cdots ,C_{t}\right\}$ , where t denotes the number of obtained dense subgraphs, we define the overlap degree of nodes of subgraph $C_{i}$ and subgraph $C_{j}\left(1\leq i,j\leq t\right)$ as $\left|C_{i}\cap C_{j}\right|/\min\;\left(\left.\left|C_{i}\right|\left.,\right|C_{j}\right|\right)$ . Simply put, it is the count of shared nodes between both $C_{i}$ and $C_{j}$ divided by the smaller of the two sets’ node counts. If the overlap degree of nodes is greater than 0.5, we merge the two subgraphs $C_{i}$ and $C_{j}$ . Additionally, during the process of obtaining a dense subgraph, we record the type to which each k -mer belongs in the subgraph, as well as the weight of a k -mer pair formed from two types of k -mer.

### 2.6 Merger and extension of k -mers

We extend the k -mers identified in the dense subgraphs obtained in the previous step. First, considering that we have recorded the type to which each k -mer belongs in the subgraph, we categorize all k -mers in each dense subgraph into two groups: enhancer k -mers and promoter k -mers. The two k -mers corresponding to the most weighted k -mer pair in each dense subgraph serve as the centers for the two types of k -mers. Next, we compare each k -mer in each type to the central k -mer, determining the position of each k -mer by assessing whether the relationship is a mismatch or an overlap. During the construction the position weight matrix (PWM), the frequency of each base corresponds to the frequency of its k -mer in the sequence. The two PWMs obtained from the dense subgraph constitute the initial DNA motif pairs. Subsequently, we use FIMO to scan the positions of the two PWMs in the real sequences. If the two PWMs appear in the sequence pair respectively, we consider them to be the final DNA motif pairs.

### 2.7 Evaluation methods for predicted DNA motif pairs

Three evaluation methods are introduced to assess the performance of the predicted DNA motif pairs. Two of these methods are utilized to evaluate the accuracy of the predicted DNA motif pairs, while the third method is employed to assess the enrichment of the predicted DNA motif pairs.

The first evaluation method is DNA motif pair distance ( MPD ), which is defined by MotifHyades and computed using Eq. 9 (Wong, 2017). The metric MPD is employed to assess how well the predicted DNA motif pairs $M=\left\{\left.\left(M_{P}^{i},M_{E}^{i}\right)\right|i\in N,i\leq K\right\}$ can be matched to the known DNA motif pairs $m=\left\{\left.\left(m_{P}^{i},m_{E}^{i}\right)\right|i\in N,i\leq K\right\}$ inserted into simulated sequence pairs:

$$MPD=\frac{1}{K}\sum_{i=1}^{K}\min_{x}\left(D\left(m_{P}^{i},M_{P}^{x}\right)+D\left(m_{E}^{i},M_{E}^{x}\right)\right),$$ (9)

where $D\left(H_{1},H_{2}\right)$ denoted the standard DNA motif distance between DNA motif $H_{1}$ and $H_{2}$ (Wong et al., 2013).

The second evaluation metric is DNA motif pair found ratio ( MPFR ), which is computed by Eq. 10 and used to estimate how many statistically significant DNA motif pairs are found correctly. A DNA motif $H_{1}$ is deemed a statistically significant ( p<0.005 ) match to another DNA motif $H_{2}$ when the standard DNA motif distance $D\left(H_{1},H_{2}\right)$ is less than 0.5 according to the empirical distribution of random DNA motif patterns (Wong et al., 2013).

$$\text{MPFR}=\frac{1}{K}\sum_{i=1}^{K}I\left[D\left(m_{P}^{i},M_{P}^{x^{\prime }}\right)<0.5\wedge \left(m_{E}^{i},M_{E}^{x^{\prime }}\right)<0.5\right],$$ (10)

where $x^{\prime }=\arg\min_{x}\left(D\left(m_{P}^{i},M_{P}^{x}\right)+D\left(m_{E}^{i},M_{E}^{x}\right)\right)$ and $I\left[condition\right]$ is the Iverson bracket used in mathematical notation and represents logical true-or-false conditions.

The third evaluation metric involves assessing the statistical significance of the enrichment of the predicted TF pairs with known TF pairs through hypergeometric testing, as computed using Eqs 11, 12:

$$p_{value}=phyper\left(m,\frac{n\left(n-1\right)}{2},M,\frac{N\left(N-1\right)}{2}\right),$$ (11)

where,

$$phyper\left(x_{1},y_{1},x_{2},y_{2}\right)=\sum_{k=x_{1}}^{\min\left(y_{1},x_{2}\right)}\frac{y_{1}!\left(y_{2}-y_{1}\right)!x_{2}!\left(y_{2}-x_{2}\right)!}{y_{2}!k!\left(y_{1}-k\right)!\left(y_{2}-x_{2}-y_{1}+k\right)!},$$ (12)

$x_{1}$ , $y_{1}$ , $x_{2}$ and $y_{2}$ are any non-negative integers. N corresponds to the number of TFs in the STRING database, while M represents the number of TF pairs. Similarly, n and m denote the number of TFs in the predicted TF pairs and the number of predicted TF pairs, respectively.

## 3 Results

### 3.1 Benchmarking MPCHG on simulation datasets and real datasets

To assess the accuracy of predicted motif pairs, we generated a total of 9000 sets of simulated data for different parameters and computed both the DNA motif pair distance and DNA motif pair found ratio for these 9000 sets of simulated data. Additionally, to explore the biological significance of predicted motif pairs, we identified that they may contribute to chromatin interactions based on the transcription factors they bind. Finally, we compared the accuracy of predicted motifs with existing software and found MPCHG to exhibit higher accuracy.

#### 3.1.1 The DNA motif pairs predicted by MPCHG obtained high quality DNA motif pair distance and DNA motif pair found ratio on different simulation data

**FIGURE 2:**
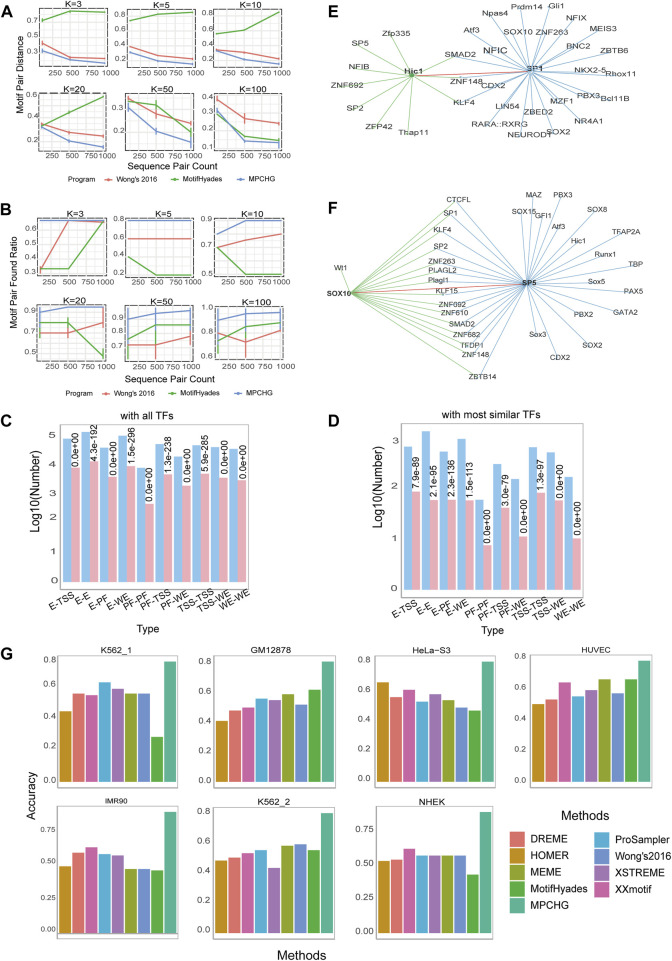
Performance of MPCHG on simulation datasets and real datasets. **(A)** Line chart for Motif Pair Distances (i.e., MPD). on known DNA motifs from JASPAR. **(B)** Line chart for Motif Pair Found Ratio (i.e., MPFR). on known DNA motifs from JASPAR. **(C,D)** Histogram on the predicted DNA motif pairs enriched with known interacting TF pairs. The red columns indicate the predicted log10 (TF pairs num) and the blue columns indicate the predicted log10 (TF pairs num supported by STRING database, which is experimentally proven). **(E)** The TF pair Hic1-SP1 in the network of the TFs corresponding to the predicted DNA motifs in K562_1 cell line. The green line represents the TFs interacting with TF Hic1 and the blue line represents the TFs interacting with TF SP1. The red line indicates the interaction between Hic1 and SP1, leaving out some of the lines between the interacting TFs. **(F)** The TF pair SOX10-SP5 in the network of the TFs corresponding to the predicted DNA motifs in HUVEC cell line. The green line represents the TFs interacting with TF SOX10 and the blue line represents the TFs interacting with TF SP5. The red line indicates the interaction between SOX10 and SP5, leaving out some of the lines between the interacting TFs. **(G)** Histogram of the accuracy comparison between MPCHG and other six methods on seven datasets. The horizontal axis represents different methods, while the vertical axis indicates the accuracy of predicted DNA motifs.

We generate 9000 sets of simulation data to evaluate the performance of MPCHG. The simulated sequences follow a Gaussian distribution with a mean of 500 nucleotides and standard deviation of 20 nucleotides for basic benchmarking. The number of DNA sequence pairs T) is varied from 100 to 1000. Subsequently, we randomly select H DNA motif profile matrices from the JASPAR database, and the number of DNA motif pairs is varied from 3 to 100 through random combinations. We select the base-generating string corresponding to the number with the highest probability based on the distribution of bases at each position within each profile matrix. Afterward, we randomly replace the selected strings in the sequence pairs with the generated string pairs. The complete performance spectrum is visualized in Figure 2A and Figure 2B. The DNA motif pairs identified by MPCHG consistently exhibited high-quality DNA motif pair distances and DNA motif pair found ratios across diverse simulation datasets. In addition, we can see from Figure 2A and Figure 2B that with more sequence pairs, MPD decreases while MPFR increases, suggesting MPCHG’s better generalization on larger datasets and its potential for enhanced robustness, leading to more reliable and accurate predictions.

#### 3.1.2 The interacting TF pairs obtained by predicted DNA motif pairs were significantly enriched with known interacting TF pairs and the TF pairs obtained by predicted DNA motif pairs may contribute to chromatin interactions

It is widely recognized that the interaction between DNA motifs is facilitated by transcription factors (TFs). Thus, we predict TF interactions based on the interactions between DNA motifs (Yu et al., 2006). Utilizing the JASPAR database, we can retrieve information about which TFs bind to each DNA motif. Subsequently, we compare the predicted DNA motifs with DNA motifs in the JASPAR (NON-REDUNDANT) DNA-JASPAR CORE (2022) vertebrates database to identify the TFs associated with the predicted DNA motifs. Based on the interactions between DNA motifs and the TFs bound by each DNA motif, we derive TF pairs. During the process of obtaining TF pairs from DNA motif pairs, it is noteworthy that a DNA motif may bind to multiple TFs. Therefore, we consider two approaches for the TFs associated with a predicted DNA motif: one involves including all TFs for the predicted DNA motif, while the other involves considering only 1 TF. For a given DNA motif, we first identify the most similar DNA motif in the database, i.e., the DNA motif corresponding to the lowest p_value . Subsequently, we designate the TF of this most similar DNA motif as the TF of our predicted DNA motif. This yields two types of TF pairs corresponding to predicted DNA motif pairs.

To assess whether the predicted TF pairs are enriched with known TF pairs, we collect experimentally validated interacting TFs in STRING database (Szklarczyk et al., 2023). Then, we use hypergeometric testing to calculate the $p_{value}$ , evaluating the statistical significance of the enrichment of the predicted TF pairs with known TF pairs. The findings for the K562_1 cell line are illustrated in Figure 2C and Figure 2D, while the results for the remaining 6 cell lines are presented in Supplementary Figures S2A, S2B. These figures unveil a notable and statistically significant enrichment of the predicted interacting transcription factor (TF) pairs with the established TF interactions in the STRING database.

The TF pairs we have predicted are likely to play a role in chromatin interactions. To illustrate, by comparing our predicted TF pairs with experimentally validated TF pairs in the STRING database, we identify a novel predicted TF pair, HIC1-SP1, as depicted in Figure 2E. HIC1 is a transcription factor (TF) classified as a member of the BTB/POZ (Broad complex, Tramtrack, Bric à brac or poxvirus and zinc finger) zinc finger family. These TFs are characterized by the presence of an N-terminal POZ domain involved in protein-protein interactions and a C-terminal zinc-finger binding domain for direct DNA interaction. A recent report reveals that HIC1 can act as both a transcriptional repressor and an activator during induction of human regulatory T cells (Ray and Chang, 2020). SP1, also known as specificity protein 1*, is a protein that in humans is encoded by the SP1 gene. The protein encoded by this gene is a zinc finger transcription factor that binds to GC-rich DNA motifs of many promoters (Al-Sarraj et al., 2005). Notably, Hypoxia repressed SIRT1 transcription through promoting the competition between Sp1 and HIC1 on the SIRT1 proximal promoter in a SUMOylation-dependent manner (Sun et al., 2013). Based on this, the competitive relationship between SP1 and HIC1 may regulate gene transcription by influencing chromatin structure and status. Furthermore, another novel DNA motif pair, SOX10-SP5, as illustrated in Figure 2F, is predicted in HUVEC cell line. Sox10 is present in all neural crest cells and plays a particularly vital role in determining the fate, viability, and maturation of Schwann cells originating from neural crest stem cells (Mao et al., 2014). SP5 binds to the GC box, a DNA motif present in the promoter of a very large number of genes (Harrison et al., 2000), and is an essential early regulator of neural crest specification in *xenopus* (Park et al., 2013). Furthermore, experimentally validated by Choi et al. demonstrated that knocking down Sp5 on the initial steps of neural crest development could result in complete loss or reduction of the expression of NC markers Sox10 (Park et al., 2013). Thus, it is likely that the interaction of SOX10-SP5 contributes to chromatin interactions, allowing their transcripts to co-localize in the neural crest region (Park et al., 2013).

#### 3.1.3 MPCHG achieved a higher accuracy than existing methods in identifying DNA motifs

We finally assess the accuracy of the DNA motifs obtained in the intermediate process to understand the degree of overlap with existing DNA motifs. We conduct a comparative analysis of MPCHG against six state-of-the-art DNA motif-finding tools, namely, DREME (Bailey, 2011), HOMER (Heinz et al., 2010), MEME (Bailey et al., 2006), ProSampler (Li et al., 2019), XSTREME (Grant and Bailey, 2021), and XXmotif (Hartmann et al., 2013). All these tools utilize the JASPAR database as a reference and employed the TomTom software (Gupta et al., 2007) with default parameters for assessment. In particular, MEME requires user to specify the number of DNA motifs, and after systematic testing at output settings of 50, 100, 150, and 200 DNA motifs, the optimal parameter of 50 is determined (yielding the highest accuracy in comparison with the JASPAR database). The remaining parameters of the above-mentioned algorithm are set to their default values, the accuracy of each method can be observed in Figure 2G. The results show that the accuracy of motifs predicted by MPCHG on 7 sets of data ranges from 75.0% to 88.7%. In contrast, the accuracy of the other six methods range from 28.0% to 66.7%. Where, MotifHyades exhibits the lowest accuracy at 28.0% on K562_1 cell line. Overall, MPCHG demonstrates an improvement of around 60% in accuracy compared to the other six methods. Notably, the accuracy of MPCHG averaged around 80% across various cell lines, indicating its high robustness.

### 3.2 DNA motif spatial accessibility and functional correlations provide insights into predicted DNA motif pairs

Exploring the spatial accessibility of motif pairs can unveil their mechanisms of action in gene regulation. By assessing the spatial accessibility of these motif pairs, we can determine which gene regions are more prone to transcription factor binding, thus gaining deeper insights into key nodes within the gene regulatory network. Furthermore, investigating the functional correlations between motif pairs can reveal their synergistic roles and functional regulations in biological processes, thereby understanding their functions and regulatory mechanisms in specific biological processes.

#### 3.2.1 DNA motifs predicted by MPCHG are spatial accessible and DNase peak fractions of different type of DNA motifs have different correlation to the number of enriched GO terms

**FIGURE 3:**
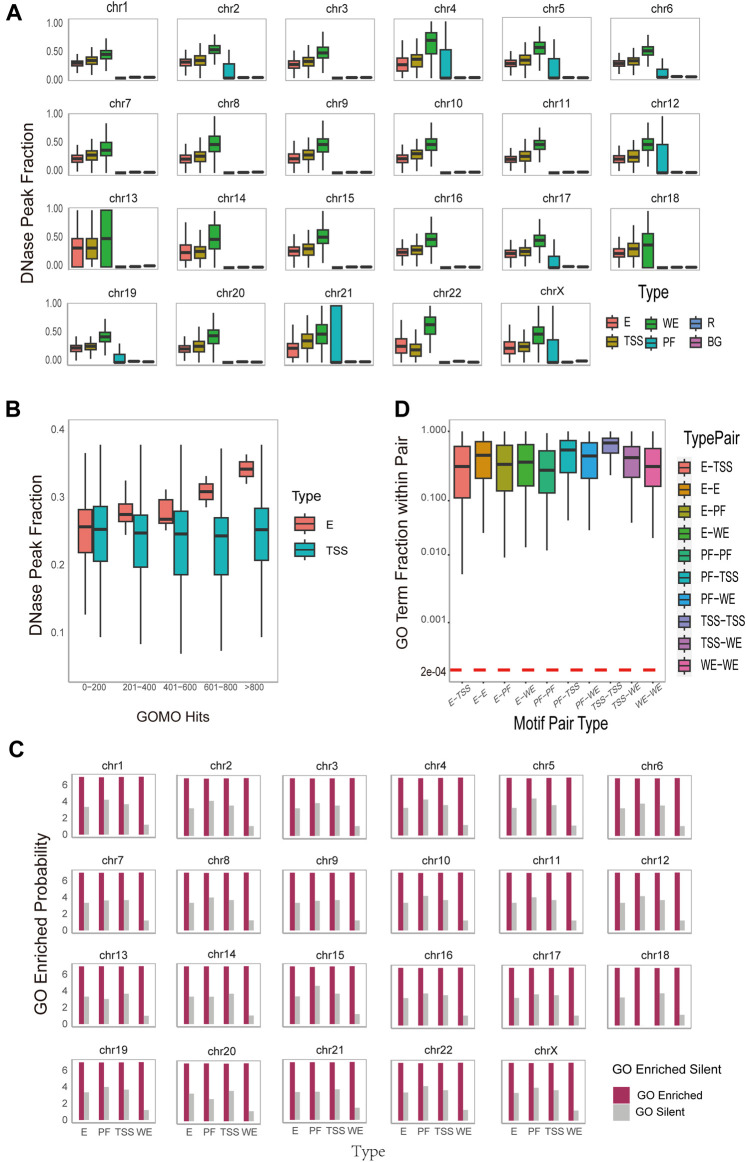
Functional and Spatial-level analysis of identified motif pairs. **(A)**. Box plots on the DNase hypersensitivity peak fraction of the DNA motifs found on different region types (i.e., WE (Weak Enhancer), E (Enhancer), TSS (Promoter), PF (Promoter-Flanking Region), R (Regulatory Region Background), BG (Background)) on different chromosomes. The horizontal axis represents different type of DNA motifs, while the vertical axis, DNase Peak Fraction, represents the ratio of the number of DNA motifs that overlap with DNase hypersensitive sites to the total DNA motifs. **(B)**. Box plots on the DNase hypersensitivity peak fraction of the DNA motifs found on different region (i.e., E (Enhancer) and TSS (Promoter)) with varying numbers of enriched Gene Ontology (GO) terms. **(C)**. Histogram of GOMO gene ontology enrichment results, with DNA motifs identified and sorted by type (horizontal axis), and the vertical axis is converted to 7+log(probability the proportion of DNA motifs with at least one GO term in each type). For each DNA motif, the term “GO Enriched” indicates that it has at least one statistically significant GO term identified by GOMO, while the term “Silent” indicates that there is no statistically significant GO term identified by GOMO. **(D)**. Boxplot on the overlap coefficients (Szymkiewicz-Simpson coefficients) between the enriched GO terms of the first DNA motif and those of the second DNA motif within each DNA motif pair. The arrangement is sorted by type on the horizontal axis. A horizontal red dashed line serves as a reference for the expected overlap coefficient under the null hypothesis. This assumption posits that the overlap is entirely random, featuring a uniform hit distribution for all identified GO terms in this study conducted by GOMO.

Exploring the accessibility of DNA motifs is instrumental in identifying gene regions prone to transcription factor binding, offering insights into the underlying mechanisms of gene regulation. To investigate DNA motifs accessibility, we download the DNase Chip-seq peak-calling data (Supplementary Table S1) from the ENCODE consortium (Dunham et al., 2012) across 6 cell lines. We calculate how many DNA motifs overlap with DNase hypersensitive sites on the reference hg19 human genome. To measure the significance of DNase Peak Fraction, we adopt the approach used by Wong in 2016 (Wong et al., 2016). For each DNA motif instance, we randomly sample 100 sites of the same width from both the regulatory region and the entire region of the same chromosome. This process yields regulatory region background DNase peak fractions (denoted as R) and overall background DNase peak fractions (denoted as BG) for each chromosome, respectively. The result (Figure 3A) for K562_1 cell line and the result (Supplementary Figures S3–S8) for other 6 cell line illustrate the DNase Peak Fraction for different types of DNA motifs on each chromosome individually. As depicted in the Figure 3A, the DNase Peak Fraction consistently follows a pattern across various DNA motif types: WE motifs exhibit the highest DNase Peak Fraction, followed by TSS motifs, and the lowest PF, except for the 22nd chromosome. E motifs and TSS motifs have relatively almost the same size DNase Peak Fraction, but both are higher than R and BG motifs. This suggests that WE motifs are more inclined to be open, followed by TSS motifs, and this overlapping fraction is statistically significant. We conduct t-tests and Mann-Whitney tests to measure the statistical significance of the difference between the identified DNA motifs and those in the background region. The result indicates that all p−values are less than 0.01, signifying a significant overlap between DNA motifs predicted by our method and DNase hypersensitive sites.

Furthermore, DNase peak fractions exhibit distinct correlations with the number of enriched GO terms for different type of DNA motifs. As illustrated in Figure 3B, for enhancer motifs, the DNase peak fraction displays a positively correlation with the number of enriched GO terms, while for TSS motifs, it remains almost unchanged. This observation may be attributed to the fact that enhancers, responsible for gene expression regulation, are typically located in open chromatin regions known as DNase hypersensitive sites. These sites, susceptible to nucleases like DNase I, represent chromatin regions that are not tightly bound in the nucleus, allowing easier access to DNA structures by regulatory elements such as transcription factors. Consequently, the increase in the number of GO terms associated with enhancer motif enrichment and their overlap ratio with DNase hypersensitive sites may be attributed to the likelihood of these enhancers being situated in open chromatin regions. This accessibility facilitates interactions with regulators, influencing the enrichment of GO terms. On the other hand, TSS motifs are commonly found in the promoter region of a gene, associated with the transcription start site. While these motifs play a crucial role in gene initiation, an increase in their number does not lead to a significant change in the overlap ratio with TSS. This is because the location of TSS motifs in the promoter region is relatively fixed, and there is no direct correlation with an increase in the number of GO terms. Despite the increase in enriched GO terms for enhancer motifs, these terms do not directly impact the distribution of TSS motifs. Therefore, the overlap ratio with TSS remains largely unchanged. This phenomenon underscores the importance of distinguishing between various regulatory elements and factors in the study of gene regulation. It emphasizes the necessity of considering their intricate interactions within the gene expression regulatory network.

#### 3.2.2 DNA motifs predicted by MPCHG are enriched with GO terms and the two DNA motifs coupled within one DNA motif pair are functional consistency

Ontology enrichment analysis serves as a crucial bioinformatics tool, facilitating the identification of significant enrichment in a group of genes or gene-associated entities in biological functions and processes (Peng et al., 2019). This analysis provides comprehensive insights into the functional characteristics of the study subject, shedding light on its significant roles in biology. To conduct this analysis, we use GOMO software for Gene Ontology enrichment on each DNA motif obtained (Buske et al., 2010). In short, GOMO scans all promoters using the provided DNA motifs to determine if any DNA motif is significantly associated with genes linked to one or more Gene Ontology (GO) terms. This process is significant for understanding the biological roles of the DNA motifs. The results are depicted in Figure 3C and Supplementary Figures S9–S14. Notably, on average, more than 97% of DNA motifs exhibit enrichment for at least one GO term. This observation suggests that the predicted DNA motifs play a discernible role in gene regulation, cellular processes, or other biological functions, and their functions may be relatively extensive and universal. Among the top frequent terms, we observe the DNA motifs-related GO terms such as (GO:0048731 system development) (GO:0048513 animal organ development), (GO:0030154 cell differentiation), and (GO:0003700 DNA-binding transcription factor activity).

Furthermore, our interest extends to the functional roles between the two DNA motifs within each DNA motif pair. To explore this, we calculate the overlap coefficient (Szymkiewicz-Simpson coefficient) between the enriched GO terms of the first DNA motif and those of the second DNA motif within each DNA motif pair. The results of the overlap coefficient are illustrated in Figure 3D and Supplementary Figure S15. The observed overlap coefficients are higher than expected, indicating a substantial overlap between the two DNA motif-related GO term set. This suggests a potential functional or biological correlation between the two motifs. Notably, the overlap coefficient for TSS-TSS interaction is the highest, implying that interactions between promoters may be functionally more closely related, involved in more common biological processes, and exhibit stronger functional correlations. These findings provide valuable insights for a deeper understanding of promoter interaction in gene regulatory network and biological processes. Additionally, they offer guidance for further functional annotation and research into regulatory mechanisms.

### 3.3 DNA motif pairs predicted by MPCHG unveiled genomic distance characteristics in human cell lines

To analyze genomic distance signatures within chromatin structures, we first counted the motif pairs predicted by MPCHG on seven cell lines. Through genomic distance analysis of the predicted motif pairs, MPCHG reveals the spatial relationships and interactions between the regulatory elements. Notably, these findings highlight the universality of long-distance regulatory mechanisms, and in particular enhancers play a key role in facilitating precise gene regulation.

#### 3.3.1 DNA motif pairs were discovered by MPCHG on seven human cell lines

**FIGURE 4:**
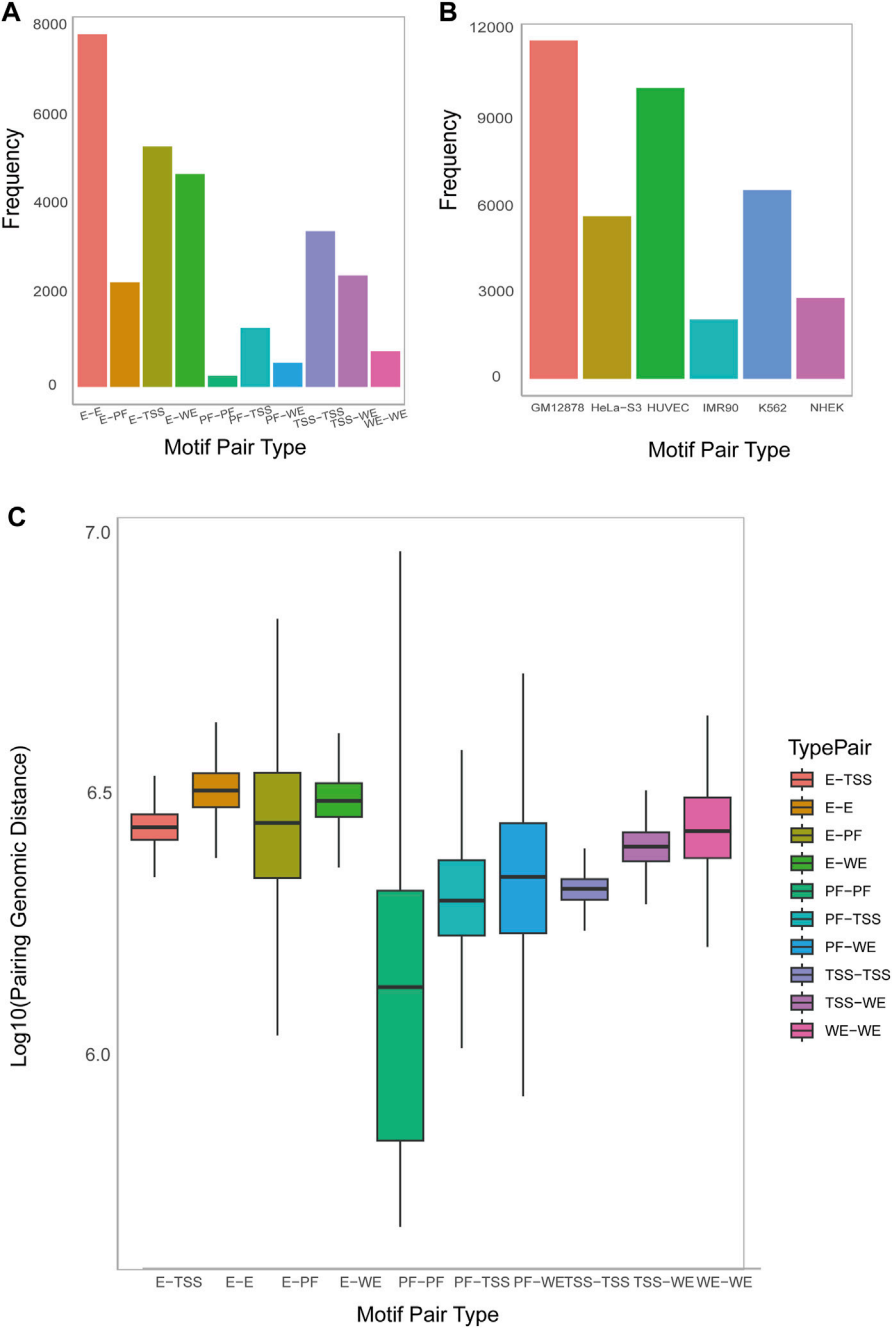
The num and spatial distribution relationship of identified DNA motif pairs. **(A)**. Histogram on the number of predicted motif pairs annotated to different types by ChromHMM and Segway (i.e., E (Enhancer), WE (Weak Enhancer), TSS (Promoter), and PF (Promoter-Flanking Region)). **(B)**. Histogram on the number of predicted motif pairs annotated by six cell lines. Their chromatin interaction type is exclusively Promoter-Enhancer. **(C)**. Boxplot on the average genomic distance between the motif instances of the first DNA motif and those of the second DNA motif within each DNA motif pair, sorted by type (horizontal axis).

MPCHG has run on the seven cell lines (K562_1, GM12878, HeLa-S3, HUVEC, IMR90, K562_2, NHEK) to obtain ten thousand of DNA motif pairs. We counted the number of DNA motif pairs of 10 chromatin interaction types on the K562_1 cell line and the number of promoter-enhancer-pairs on the remaining six cell lines. The discovered DNA motif pairs are visualized Figure 4A and Figure 4B.

#### 3.3.2 The genomic distance between DNA motifs pairs predicted by MPCHG revealed the interaction and relative position between the regulatory elements

Analyzing the distances between DNA motifs provides insights into the relative positioning and interactions of gene regulatory elements, indicating whether they are in close proximity or distantly located within the three-dimensional chromatin structure (Dekker and Misteli, 2015). This analysis enhances our understanding of the organization and spatial regulation of gene expression at the chromatin level. Therefore, Accordingly, we have computed the distance between DNA motifs of different interaction types. As depicted in Figure 4C, the interaction distance between E-E is the greatest, followed by E-WE, E-TSS, and E-PF and Supplementary Figure S16 also indicate that enhancers are far away from Promoters. This observation aligns with the widely accepted notion that enhancers are typically situated in regions far away from the genes they regulate, sometimes spanning millions of base pairs (bp). This long-distance regulatory action is facilitated through the establishment of chromatin loops, enabling effective and precise regulatory interactions.

## 4 Discussion

Identifying DNA motifs is of paramount importance in biology and computational biology. DNA motifs are short sequence patterns in protein or nucleic acid sequences that are functionally relevant. They are crucial for functional annotation, structure prediction, evolutionary relationships, and regulatory element recognition. Furthermore, the identification of DNA motif pairs in interacting sequences is also significant as it aids in predicting protein-protein interactions, drug design, and disease research. In conclusion, DNA motifs and their pairs play pivotal roles in biological research and medical applications.

Hence, we propose the MPCHG algorithm to identify tens of thousands of DNA motif pairs in the long-range chromatin interaction sequences. First, we use a 3-order Markov model to generate background sequences that matches the length and composition of the original sequence, ensuring statistical significance and rationality for k -mer seeds. In contrast to many algorithms that exhaustively determine DNA motif length within a specific range, our method extends the core DNA motif to both ends using a double-sample z-test. This approach aligns the predicted DNA motif more closely with real scenarios. At the same time, algorithms that set the DNA motif length in advance may miss some important DNA motifs or introduce high noise. Furthermore, we construct a composite heterogeneous graph for different types of k -mers (enhancer k -mers from enhancer sequences and promoter k -mers from promoter sequences). This graph connects k -mers of different types present in the same sequence pair. Simultaneously, it captures complex relationships among k -mers of the same type with mismatched or overlapping connections. To obtain a dense subgraph related to k -mers, we define the fitness function of the subgraph to assess its density. Nodes meeting specific conditions are extended to the current seed. Finally, we merge and extend the obtained subgraph to extract DNA motifs. Subsequently DNA motifs scanning enables the identification of DNA motif pairs.

Regarding the predicted DNA motif pairs, we conducted a thorough analysis covering various aspects. The accuracy rate, measured by comparing predicted DNA motifs with the JASPAR database using TOMTOM software, the accuracy of predicted DNA motifs ranged from 75.0% to 88.7%. Additionally, we employed GOMO software to explore the gene ontology enrichment of these predicted DNA motifs. The findings revealed that, on average, over 97% of DNA motifs are enriched for at least one GO term. This indicated that the predicted DNA motif play essential roles in gene regulation, cellular processes, or other biological functions, and their functions may be relatively extensive and universal. To further validate our predictions, we compare the predicted DNA motifs with DNase Chip-seq peak-calling data. The analysis demonstrated a significant overlap between DNA motifs predicted by our model and DNase hypersensitive sites. Notably, DNA motif pairs involving enhancer or weak enhancer regions exhibited greater distance, aligning with the common understanding that regulatory components in enhancer regions are typically located far from their interacting partners, often spanning a large genomic distance. We extended our analysis to predict TF interactions based on the predicted DNA motif pairs. The result indicated that the predicted interacting TF pairs are significantly enriched with the known interacting TF pairs in STRING, as determined by hypergeometric testing. Moreover, we unveiled new TF interaction information, such as the interaction between HIC1 and SP1, suggesting a potential role in facilitating chromatin interactions and promoting gene transcription. Finally, to evaluate the generalization performance of our model, we tested it on six additional E-TSS datasets representing different cell lines (GM12878, HeLa-S3, HUVEC, IMR90, K562, and NHEK). The results demonstrated consistently good performance across these diverse datasets.

The prediction of DNA motif pairs stands as a critical challenge in bioinformatics, offering valuable insights into various biological processes, including gene regulation, protein-protein interactions, and RNA structures. While significant strides have been made in this field, the future holds immense potential for further advancements. Firstly, the continuous evolution of deep learning and artificial intelligence techniques, including innovative algorithms and graph neural networks, is expected to elevate the accuracy and reliability of DNA motif pair predictions. Secondly, the exploration of cross-species DNA motif pair prediction presents an intriguing challenge, offering opportunities to uncover conserved sequence patterns and explore evolutionary variations. Thirdly, the integration of diverse data sources, such as epigenetic data and protein interaction information, will contribute to more comprehensive annotations for predicted results, enhancing our understanding of the intricacies of biological systems. Additionally, applying DNA motif pair predictions in disease research and precision medicine holds promise for identifying potential disease markers or therapeutic targets. Lastly, the combination of DNA motif pair predictions with network interactions and systems biology approaches will enable the construction of comprehensive biological regulatory network models. This integrative approach has the potential to deepen our understanding of the fundamental principles of biology. In conclusion, ongoing research in predicting DNA motif pairs has significant potential to drive breakthroughs in biotechnology and medical advancements, fostering progress in the fields of biology and medicine.

## Data Availability

Original datasets are available in a publicly accessible repository: The original contributions presented in the study are publicly available. The input data of Hi-C data for six cell lines (K562, GM12878, HeLa-S3, HUVEC, IMR90, and NHEK) can be found here: https://doi.org/10.6084/m9.figshare.14192000. The protein-protein interaction data from the STRING database can be found here: https://cn.string-db.org/cgi/download?sessionId=blEDX7XXpqWC.
