# Supplementary material for: Identification of DNA motif pairs on paired sequences based on composite heterogeneous graph
Source: Front Genet. 2024 Jun 17;15:1424085. doi: 10.3389/fgene.2024.1424085 (PMC11215013; doi:10.3389/fgene.2024.1424085)
Supplement: Supplementary file 1 [file DataSheet1.ZIP › Supplementary_Material.docx]

Supplementary Material

# 1 Supplementary Figures and Tables

## 1.1 Supplementary Figures

**Supplementary Figure 1.** Histogram on the number of different cell lines. **(A).** Histogram on the number of long-range regulatory region pairs annotated to different types by ChromHMM and Segway (i.e. E (Enhancer), WE (Weak Enhancer), TSS (Promoter), and PF (Promoter-Flanking Region)). **(B).** Histogram on the number of regulatory region pairs for six cell lines. Their chromatin interaction type is exclusively Promoter-Enhancer.

**Supplementary Figure 2.** Histogram on the predicted motif pairs are enriched with known interacting TF pairs for six cell lines (GM12878, HeLa-S3, HUVEC, IMR90, K562, NHEK). The red columns indicate that the predicted log10(TF pairs num) and the blue columns indicate that the predicted log10(TF pairs num supported by STRING database which is experimentally proven). **(A).** Histograms showcase the enrichment of predicted motif pairs with known interacting TF pairs when considering all TFs for each given motif. **(B).** Histograms demonstrate the enrichment of predicted motif pairs with known interacting TF pairs when considering only one TF for each given motif.

**Supplementary Figure 3.** Box plots on the DNase hypersensitivity peak fraction of the DNA motifs found for GM12878 cell lines on different chromosomes.

**Supplementary Figure 4.** Box plots on the DNase hypersensitivity peak fraction of the DNA motifs found for HeLa-S3 cell lines on different chromosomes.

**Supplementary Figure 5.** Box plots on the DNase hypersensitivity peak fraction of the DNA motifs found for HUVEC cell lines on different chromosomes.

**Supplementary Figure 6.** Box plots on the DNase hypersensitivity peak fraction of the DNA motifs found for IMR90 cell lines on different chromosomes.

**Supplementary Figure 7.** Box plots on the DNase hypersensitivity peak fraction of the DNA motifs found for K562 cell lines on different chromosomes.

**Supplementary Figure 8.** Box plots on the DNase hypersensitivity peak fraction of the DNA motifs found for NHEK cell lines on different chromosomes.

**Supplementary Figure 9.** Histogram on the GOMO gene ontology enrichment results for GM12878 cell line. Horizontal axis denotes the identified DNA motifs sorted by chromosomes and vertical axis denote the proportion of motifs containing at least one Gene Ontology term to the total number of motifs of each chromosome. Each motif labeled as “GO Enriched” indicates it possesses at least one statistically significant GO term identified by GOMO and “GO Silent” means it has not GO term identified by GOMO.

**Supplementary Figure 10.** Histogram on the GOMO gene ontology enrichment results for HeLa-S3 cell line. Horizontal axis denotes the identified DNA motifs sorted by chromosomes and vertical axis denote the proportion of motifs containing at least one Gene Ontology term to the total number of motifs of each chromosome. Each motif labeled as “GO Enriched” indicates it possesses at least one statistically significant GO term identified by GOMO and “GO Silent” means it has not GO term identified by GOMO.

**Supplementary Figure 11.** Histogram on the GOMO gene ontology enrichment results for HUVEC cell line. Horizontal axis denotes the identified DNA motifs sorted by chromosomes and vertical axis denote the proportion of motifs containing at least one Gene Ontology term to the total number of motifs of each chromosome. Each motif labeled as “GO Enriched” indicates it possesses at least one statistically significant GO term identified by GOMO and “GO Silent” means it has not GO term identified by GOMO.

**Supplementary Figure 12.** Histogram on the GOMO gene ontology enrichment results for IMR90 cell line. Horizontal axis denotes the identified DNA motifs sorted by chromosomes and vertical axis denote the proportion of motifs containing at least one Gene Ontology term to the total number of motifs of each chromosome. Each motif labeled as “GO Enriched” indicates it possesses at least one statistically significant GO term identified by GOMO and “GO Silent” means it has not GO term identified by GOMO.

**Supplementary Figure 13.** Histogram on the GOMO gene ontology enrichment results for K562 cell line. Horizontal axis denotes the identified DNA motifs sorted by chromosomes and vertical axis denote the proportion of motifs containing at least one Gene Ontology term to the total number of motifs of each chromosome. Each motif labeled as “GO Enriched” indicates it possesses at least one statistically significant GO term identified by GOMO and “GO Silent” means it has not GO term identified by GOMO.

**Supplementary Figure 14.** Histogram on the GOMO gene ontology enrichment results for NHEK cell line. Horizontal axis denotes the identified DNA motifs sorted by chromosomes and vertical axis denote the proportion of motifs containing at least one Gene Ontology term to the total number of motifs of each chromosome. Each motif labeled as “GO Enriched” indicates it possesses at least one statistically significant GO term identified by GOMO and “GO Silent” means it has not GO term identified by GOMO.

**Supplementary Figure 15.** Boxplot on the overlap coefficients (Szymkiewicz-Simpson coefficients) between the enriched GO terms of the first motif and those of the second motif within each motif pair sorted by cell line (horizontal axis). The horizontal red dashed line indicates the expected overlap coefficient under the null hypothesis assumption that the overlap is purely random with the uniform hit distribution for all the GO terms identified in this study by GOMO.

**Supplementary Figure 16.** Boxplot on the average genomic distance between the motif instances of the first DNA motif and those of the second DNA motif within each DNA motif pair, sorted by cell lines (horizontal axis).

## 1.2 Supplementary Table

**Table 1:** List of DNase Peak File used from the ENCODE project.

| DNase Peak File | Num | Average Length |
| --- | --- | --- |
| wgEncodeUWDukeDnaseGM12878.fdr01peaks.hg19.bb | 183953 | 151 |
| wgEncodeDukeDnaseHeLaS3IFNa4h.fdr01peaks.hg19.bb | 117383 | 151 |
| wgEncodeUWDukeDnaseHUVEC.fdr01peaks.hg19.bb | 194101 | 151 |
| wgEncodeDukeDnaseIMR90ENCFF000STJ.bb | 163343 | 207 |
| wgEncodeUWDukeDnaseK562.fdr01peaks.hg19.bb | 202266 | 151 |
| wgEncodeUWDukeDnaseNHEK.fdr01peaks.hg19.bb | 214369 | 151 |
